# Annual Report of the Museum of Hygiene, United States Navy

**Published:** 1887-03

**Authors:** 

**Affiliations:** Medical Director, U. S. N., in charge


### ANNUAL REPORT OF THE MUSEUM OF HYGIENE.

ANNUAL REPORT OF THE MUSEUM OF HYGIENE OF THE UNITED STATES NAVY. *By* THOMAS J. TURNER, *A.M.*, *M.D.*, *Ph.D.*, *Medical Director*, *U. S. N.*, *in charge.*

In presenting this report upon the Museum of Hygiene, it is to be stated that the remarks of my predecessor, Medical Director John M. Browne, U. S. N., as to the necessities of the museum, apply with increased force.

Since the last report the collection of sanitary and hygienic appliances has been increased by donation and purchase until there is now insufficient room for the proper presentation of the general exhibit, either for purposes of study or inspection. The like remarks apply to the library. The shelving is now full, and the constant addition of books, pamphlets, circulars, etc., upon hygiene, both general and special, has rendered it necessary to store these accessions upon the floor space. The library, from this fact alone, is becoming valueless for reference and study, and its usefulness is consequently impaired. The catalogue, still incomplete, now numbers over fourteen thousand titles. The great number of valuable publications, and more especially the recent literature of subjects pertaining to hygiene, are not available for study or reference because unbound. The necessity for an appropriation for binding needs no other demonstration. These facts are to be taken into consideration in connection with the circumstance that there has not been a day without some call for information either from plumbers, architects, medical men, or others interested in the means of preventing dangers to health.

The necessity for a building for the objects of the museum is apparent to any observer, and it is respectfully urged that the attention of Congress be called to this condition of affairs. Other countries have established institutions of like character, and the progress of sanitation in the United States, as presented in this museum, is creditable alike to the originality of the presented inventions and the object sought to be obtained. Over thirty cases of models representing the baths at Bath, England, which formed a prominent exhibit at the late International Sanitary Exhibition, are now stored in the cellar. These models have been presented to the museum.

The apparatus of Galton for anthropometry is stored, and cannot be used for want of room. The attic is also crowded with unbroken packages. It is the opinion of those well informed upon such matters, after an examination of the present display, that it is overcrowded, and that twice the present area is necessary for proper presentation of the appliances, models, etc. There is need for a permanent clerk for the museum. The clerical duties in cataloguing, indexing, labeling, etc., utilizes most of the time of the medical officers attached to the museum, and there is but little time left for the pursuit of scientific study.

The changing of medical officers causes irregularity, and both system and method are subjected to too great variations by such changes. The officer leaving does not, nor cannot, leave his acquired knowledge to his successor, and a new arrival has of necessity the accumulated "dead work" to embarrass and obstruct his usefulness, and he, too, just as becoming efficient, is detached and ordered to his more legitimate duty at sea, and so on again and again. The advantages of the museum to original investigators and their investigations into matters connected with public health and the progress of preventive medicine are at present at their minimum for want of working room. It is respectfully suggested that the number of officers on duty at the museum be increased, preference being given to those officers who have displayed interest in and are ready to work upon matters so important to the state as public health. The tools are here, but the workmen are not now on hand.

In the physical department attempts have been made to define a standard by which to measure the relative viscosity of oils for lubricating purposes at determined temperatures, and also in colormetry as giving data for accurate measurements by instruments of precision.

The biological department has not been laid under service, but it is proposed to enter upon examinations in this direction, especially into the products made by the bacilli—the ptomaines and leucomaines. It is considered by the writer that these substances all belong to the ammonium series and are the exponents of the inverse metabolism of the amines, all tending to the ultimate isolation of nitrogen from animal organic compounds. It is also considered that the alkaloids existing in the vegetable kingdom may be found to be the result of cell action in such vegetable structures, closely allied to the action of the bacilli in animal matters. Should these projections become demonstrated facts their value, both in curative as well as in preventive medicine, can readily be made plain. The toxic phenomena produced by these substances, both from the vegetable and animal kingdoms, appear to be alike when introduced into the system, and the same phenomena occur and appear in cases of uræmic intoxication from the retention of urea—a typical example of this class of bodies

—as well as in typhus-fever where the glass rod moistened by $HC_1$ and held in the expired air gives the reaction of the gaseous amines. It may be that the odors that characterize some diseases have a like origin.*

The comparison of the poisonous effects of the vegetable alkaloids with the results of toxic effects of the various ptomaines and leucomaines, when introduced into the system, exhibit the fact that they are generically alike—nerve sedatives—narcosis marking one species, cardiac depression another, the functions of the spinal cord being affected in a third, and so on. The alkaline condition of the discharges in cholera, and their innocuousness when rendered acid, the acid treatment in typhoid-fever and the use ot disinfectants in destroying the germ and its products, are all connected with this subject. The production of these alkaloidal bodies in the living body in health, as well as in disease, is now well determined.† Their elimination in normal physiological conditions; their retention as perverting these conditions; their causative agency in the production of disease in the body in which they are formed and in the bodies of others subjected to their action, are now the subjects of extended and careful research. The influence the results of such studies must have on the future of both sanitation and therapeutic medicine for good cannot be now calculated. The further metamorphosis of these amine com-

* As this class of bodies is likely to be increased by future research, the name for the whole genera, it is hazarded, should be micro-bamines, as expressing alike origin and composition.

† It may here be mentioned that the presence of these substances in animal products, used as foods, presents a further field of investigation to the biological chemist. The discovery of a poisonous alkaloidal body in cheese and in milk, after being kept for some time, by Professor V. C. Vaughan, are but the initiative steps in this researce. There will occur other instances to the sanitarian, having perhaps a like solution, as causing disease-movement and suggesting preventive measures.

pounds, manufactured by the microbes, by the action of the oxygen in the air, resulting, when in the presence of alkalies, in the production of the nitrates and nitrites of the bases and water, has received an industrial application in the manufacture of potassium nitrate for technical uses, more especially in the preparation of gunpowder. Whether it would be remunerative enough to so utilize sewage by this process of nitrification is yet to be determined. The like utilization of the refuse from abattoirs, furnishing the animal nitrogenous matter with potassium carbonate, is suggested for experimentation by the chemist as being a source of supply of potassium nitrate in time of war, should other sources fail in such emergency. The microbe can be furnished by the biologist, and the "environment" necessary, such as temperature, moisture, air, and other attendant phenomena, can readily be determined by experimental investigation with scientific accuracy. In one special direction in naval hygiene is attention called to these bodies. These alkaloids all appear as the results of retrogressive metamorphosis; as the production of organized matter, which in a broadly comprehensive way we call here bacteria, and in solutions undergoing the so-called putrefactive changes. Bilge water is a putrefactive solution. A spirochæte has been found in it, and in former publications of the writer bilge water was suspected, on grounds which are now evident, to be a source of febrile movement on shipboard. Cleanliness of the bilges must be insisted upon hereafter.

It is also proposed to investigate the slow chemistry of concentrated and preserved foods in order to determine the time when their nutritive value begins to decidedly decrease and when it ceases.

CREMATION.

From the increasing literature upon this subject is inferred a growing interest in the community upon this mode of the disposal of the dead. This object, of so much sanitary importance, might soon be obtained were it not hampered by so much of the emotional element that appears inseparable from its consideration, as well as certain medico-legal restrictions which can be readily removed. It must be evident to the enlightened sense of the community that such mode of burial is demanded in such diseases as small-pox, scarlet-fever, diphtheria, and the like. There are but few models of apparatus for cremation on exhibit in the museum. There has been invented a portable crematory for use in rural districts, as well as in time of war.

MINING.

The successful adaptation of the electric light for mines and mining operations is now assured. Some experiments are on record in which it is attempted to discover how far the influence of tinted glass coverings to this light may be used in determining the presence of fire-damp, light carburetted hydrogen, the methane of the modern chemist. If these experiments are successful there will be an advance in the better hygiene of mines. The miner's old oil lamp, rendering the air impure by its products of combustion and its vile-smelling acroleine odors; the Davy lamp, with its dim, lurid light, and the carelessness with which it is handled, will be things of the past, and thus the dangers to health, as well as to life and limb, in mines be removed.

SCHOOL HYGIENE.

Without entering upon an extended notice of this section of sanitary science, leaving out for the present the consideration of the school-house, a note here appears necessary concerning the hygiene of the eyes of school children. Some German sanitary observers in this direction have condemned the dark slates now in use on physiological grounds, and on the same grounds have suggested the use of white slates with dark pencils. Taking these premises, with the results of some personal experience and observation in determining ranges of vision, as well as of the color-sense, the writer is led to suggest that a uniform "face" of type, as **antique,** be adopted for school books, and that the traditional black printing ink be abandoned for inks of neutral tints in printing such books.

This accepts a proviso that the paper remains as now—white and unglazed. How far sharpness of definition of letters can be secured by such color use the printer can determine. Further observations may support the correctness of these suggestions; that there is a strain upon the nervous apparatus of vision in the reading of books as printed at present (black on white) is an accepted fact; that this strain is a cause of asthenopia is also well made out, and that the apparatus of accommodation in the eye is affected follows as a consequence. This strain, expressed in ordinary language as "tired eyes," is relieved by letters printed on tinted paper and by colored printing inks on white or colored paper; and thus a circus poster, from these facts, ofttimes contains more refreshing reading, in a hygienic as well as a mental sense, than some newspapers or books. It needs but the simple statement, in this consideration, that glaring colors are always to be avoided.

### THE SIPHONAGE OF TRAPS.

The experiments, with the apparatus necessary for the purpose, upon the siphonage of traps which have been carried on at the Museum by Glenn Brown, A.A.I.A., have now been completed. The results of this investigation are forwarded with this report. It is an addition to the literature of this matter, and is to be placed alongside of the notes of Folsom, E. W. Bowditch, Waring, Philbrick, and others.

PREVENTION OF MALARIA.

To the list of plants proposed for use to modify or arrest the production of malaria, as in the eucalyptus and helianthus, there must now be added a water plant, the anacharis alsinastrum—the choke-pond weed natural order, hydrocharidaceæ. Its rapid growth and spread in marshes and rivers where it has been planted, and the cessation and disappearance of malarial and diarrhœal diseases formerly affecting villages and towns about such localities, have been grouped into a relationship. It is now suggested to cultivate this aquatic plant in marshy districts with a view to prevent the production of malaria. Further experiment is necessary to determine the value of this plant in this direction, but there seems to be enough evidence of such value to warrant this note.

More especially interested in naval hygiene, it is with some gratification that attention is called to its progress as exhibited in the museum and library.

AIR AND VENTILATION.

The model of the fish commission steamer Albatross, with the mode of ventilating that vessel, designed by Passed Assistant Engineer George W. Baird, U. S. N., is worthy the attention of the naval constructor. The improvements in ventilating shafts, with cowls for the ventilation of the berth decks of passenger vessels, show that the necessity for fresh air has at last become apparent to large steamship companies.

WATER.

The storage of water in iron tanks seems to leave nothing more to be desired in that direction, but the constant rusting and the chalybeate taste of the water has been considered objectionable. Filtration does not remove this taste. Some years ago it was recommended that the interior of the tanks should be prepared by the Barff process to prevent rust. It may not be out of place here to suggest that this process might be usefully applied to chains, anchors, and shot.

The aëration of water has been the cause of many patent appliances. Baird's aërator answers all purposes, and as it is attached to the distiller, the distillation, cooling, and aëration of water are completed by one process. The like is the result of the Normandy apparatus. The results of the observations of our medical officers upon water supply, as published in the reports of the surgeon-general of the navy, lead to the knowledge that there are few ports visited by our vessels of war at which the supply of potable water can be accepted for use on board ship with the confidence that no harm may result. Distilled water should be used at all times. On blockading duty no other water can be had save from an occasional rainfall, and it is no new thing to a naval sanitarian to state that such water is impure. It is again recommended that the interior of water-tanks be treated by the process mentioned to protect from rust. Of the hygienic use of distilled water it is to be noted that the absolute immunity of the squadron on the Asiatic station, in the years 1877–79, during the prevalence of epidemic cholera, and the decrease of all diseases of the bowels attended with flux, is ascribed to the use of distilled water on board of the vessels on that station.

The pollution of the sources of water for the supply of cities by sewage, garbage, etc., is constantly being referred to by sanitarians as a steady and increasing danger to health. To remove these dangers will demand more attention in the near future than is now given them. The destruction of garbage should be by fire, and in the cities a public crematory for such purposes is now a necessity. In the utilization of sewage there appears to be at present no profitable mode for such purpose. As a fertilizer its use appears to be best subserved by the process of intermittent, downward filtration in soils adapted to such usage. It may be that, failing to secure a degree of freedom from contamination that renders water "potable," the distillation and aëration of such non-usable waters by some mechanical device adapted to the culinary apparatus will be part of the future house sanitation. As to the disposal of fecal matter, we should profit by the observations and experiences of Oriental nations, and return it to the soil in such manner as to avoid contaminating water supplies.

FOOD.

The attached table from the bureau of provisions and clothing presents the United States naval ration as allowed by law, and the practical substitutes permitted. Without entering upon the detail of the value of each article as to its tissue-forming or heat-making value, it is believed to be an excellent ration. It is suggested that an extract of meat be added, to be used in connection with other animal foods after prolonged and exhaustive exertions of no matter what kind, as well as a part of the ration when salt meats are used. It is known that the addition of a meat-extract promotes the digestibility of salt food. In addition, the use of lactic acid is recommended as part of the ration. It is a natural constituent of the meat juices; is, perhaps, destroyed by cooking and diminished by salt-packing; is preferable to vinegar; has about the same commercial value, and it is proposed in this manner to restore it to the food. A re-arrangement of the meal hours on board ship is suggested. The extremes to be avoided are, first, an excess of food in a given time, as meals too frequently repeated in such time, and, second, the interval between the meals be not too long. The total amount of food taken in the twenty-four hours should be distributed in accordance with physiological laws.

In the direction of the preservation as well as conservation of articles or products used as foods there does not appear to have been any unusual activity. In general terms it may be stated that the tendency in this direction has been directed by the studies on antiseptics, and the most prominent of these articles, boric and salicylic acids, with other known articles, enter into the composition of the preservative means suggested. They are of very questionable utility in the preparation of foods for diets, no matter how valuable their antiseptic properties.

LIGHTING.

The use of the electric light on shipboard has proved of service. The vitiation of the air from the combustion of oil and candles is ended. Recurring to the oft-repeated fact of the etiolation of plants from the absence of light and the blanched appearance of miners and others whose avocations are in "darkness," will be of value to the observant medical officer to notice what effect the present system of lighting has upon the hæmatosis which furnishes the "ruddy cheek of health."

TRANSPORTATION OF THE SICK AND WOUNDED ON SHIP-BOARD.

The cot for transporting sick or wounded, designed by Medical Director A. L. Gihon, U. S. N., has so long been known and is so efficient that it requires no comment. The ambulance cot of Medical Director A. C. Gorgas, U. S. N., is also well known, as well as that of Medical Inspector H. M. Wells, U. S. N. A knit hammock, for a like purpose, is on exhibit in the Museum, designed by Thomas Graham, pensioner, U. S. N.

COOKING.

A model of the improved ship's galley, designed by William Young and in use in the service, is also on exhibit. It seems that a man-of-war's galley can only be understood by a man-of-war's man, for in all the contrivances adapted to utilize cooking space, galleys invented by sailor-men economize the space assigned to its limit. This galley, as far is known, has few defects.

DISINFECTION AND DISINFECTANTS.

Models of stoves for the purpose of disinfecting bedding, etc., at high temperatures are on exhibit in the Museum. There does not appear to have been much progress since the issue of Sternberg's essay on disinfection, by the American Public Health Association, in the scientific determination of the value of disinfectants. The journals on file contain notices of the application of numerous derivatives from the wood and coal tar products, asserted to be efficacious both as disinfectants and deodorizers. Judgment as to their real value must be suspended until further experimental research shall have placed them where they properly belong. The steam-atomizer invented some years ago by Medical Inspector H. M. Wells, U. S. N., and originally adapted for the use of carbolic acid solutions in the disinfection of vessels, is now made of materials not acted upon chemically, and could be utilized for the mercuric bichloride solutions now used for such purposes.

In the U. S. S. Dolphin 68 cubic feet per man is the berthing-space on the berth-deck. It is too small from physiological considerations; is below that given to an emigrant in the short passage across the ocean (72 cubic feet). The known ill effects of re-breathed air; of the depressed vital conditions induced by impure air from overcrowding, added to constantly wet decks; the diminution of the resisting power to disease movement; the epinose condition—in fact all these enter in the future into the etiology of disease on shipboard. This small cubic space has now become a potential factor in determining "origin in line of duty." Late published papers upon the future naval organization are marked with the idea of making the modern vessel of war do duty both as a fighting ship and as a transport, and by such means not to diminish the crew. Whatever is done in the direction of the double duty of fighting vessel and transport, it should be remembered, is done at the expense of health, and in just that diminution is the efficiency impaired. The solution of the question will come in time.

In all these considerations the fact must not be lost sight of that "the trade is War."

### LIFE-SAVING APPARATUS.

There are numerous appliances for such purposes—rafts, jackets, and mattresses—and all could be utilized in case of necessity. The Museum has many such contrivances, all of which present some advantages.

### HAMMOCKS.

There appears to have been no change in the hammock. It is to be hoped that some inventive genius may construct one that will do away with the bad position that the body has to assume while sleeping in one. More especially should such a hammock be used in the training-vessels, where the growing apprentices may be at least freed from any tendency to spinal deformities which the position during sleep in a hammock inevitably tends to produce. It is suggested in this connection that the crew be divided into three watches, for the purpose of securing the proper amount of sleep and rest. The suggestion is not new, and the present appears to be a favorable time to advance in this right direction; all experience goes for nothing if it is to be only accumulated and not used.

### FIREMEN AND COAL-HEAVERS.

Following closely upon the division of the crew into three watches for rest and sleep comes the consideration of the hygiene of the engineer's division. The new fire-rooms are to be under an increased air-pressure when the vessel is under steam. This factor of increased air-pressure is to be added to the effects of high temperature, and the results of this sum upon the health is looked for with interest. Forecasting from the known results of each factor separately upon the health of workmen, it is conjectured that, plus the exhaustion induced by the heat, there will be in some degree phenomena that occur in the "caisson" disease, and that the brunt of these influences will be evidenced by some impairment of the functions of the spinal cord. This exposure must of necessity occur, and to limit such it is suggested to divide this department into four watches, when upon duty, as affording the necessary time for a re-approach to a recovery of a normal condition.

### THE MEDICAL OFFICER ON SHIPBOARD.

It does not need any demonstration that with the increase of the efficiency of men-of-war as fighting machines, or the tactical management of troops on shore, the duties of the medical officer have vastly increased, and are out of proportion to his present position. In addition to his duties in the care of the sick and wounded, the prevention of the causes of disease in the men and the means for the preservation of their health demands an amount of practical knowledge and vigilance that belongs alone to his profession. With the political aspects of war he has nothing to do, as such; all his efforts are to conserve the health of the men, so that their strength and endurance, when the moment for fighting has come, are at their maximum and kept so. The action over, his duties are obvious, and need not be narrated. To those who have had the experience description is out of place, and to those who have not, no description can adequately detail that duty. And even in the groaning wards of his perhaps improvised hospital, his sanitary vigilance and knowledge is not to be hampered, for he recognizes, in even this assemblage of sick and wounded, enemies to the health of those around. From my own experience I have stated that the medical officer of a vessel of war should be, by law, her sanitary officer, and recent events in the ocean steamship service lead me to believe that in this respect the mercantile marine will be far in advance of the naval service. What rewards, what definite position the naval surgeon is to have in the future naval hierarchy, is at present conjectural, and will remain so until settled in some manner alike creditable to all concerned, which will only be when calm, impersonal and unselfish logical deductions are received and accepted.

HYGIENIC AND SANITARY EMERGENCIES.

Mention is made of these subjects simply to recall to the medical officer that he may be called upon to improvise means of transportation, for the shelter of sick and wounded, for hospital purposes, etc. In these matters

"The readiness is all."

The length of time that I have been upon the present duty does not permit me to give in further detail the recent progress in sanitation. Other duties, however, have not prevented the writer from noting the currents in the direction favorable to public health. A cursory survey of the stream may not prove unacceptable.

The reports of the various State Boards of Health mark the increasing interest and vigilance of physicians as guardians of public health. In each report the sanitary wants of the State obtrudes itself at once. The prominent industries in each locality finds some sanitarian making his observations for the commonweal upon its hygiene; here another keeps track of the hygiene of schools; another watches over the sanitation of the charities; there is in our country hardly an industry or charity, hospital or asylum, reformatory or prison, market or farm, house or village, or city that has not some earnest worker in preventive medicine. Not only is the physician engaged; the artisan at work upon "the preservation and conservation of foods" calls in scientific knowledge to his aid. Adulterations, as affecting public health have already secured national legislation; even the use of stimulants and narcotics has given rise to a political element in public affairs in so far as they affect the general health. Railroad traveling, on some of the prominent roads, has its ambulance system, and the traveler by sea is being cared for as regards his health and safety. The architect and engineer design and construct the works whose necessity the sanitarian has demonstrated, for drainage, the removal of excreta, the sewage, and for water-supply. The disposal of sewage and excreta is becoming daily of more concern to the agriculturist, engineer and sanitarian.

The experiments upon the deportment of soils toward human excreta in various stages of change that have been inaugurated in Japan are of extreme interest to the farmer and sanitarian, as exhibiting the disinfecting power of various soils—that is to say, in the destruction of minute organisms by the agency of earth, and also to the engineer as to the means of transportation to localities the excreta where such fertilizer is needed. The results of these investigations, as far as can be ascertained, are akin to those found by Professor R. Pumpelly for the National Board of Health, and which are to be found in the report of that body for the year 1861.

In industrial hygiene the effects of the inhalation of the dust from the manufacture of slates and slate pencils for schools has called for well-known devices to get rid of the dust in the breathing air. The lung affections caused by the inhalation of this dust need not be erected into a new disease, to be called slatemaker's asthma, bronchitis, or phthisis, for the phenomena are the same as those produced by the mechanical irritation of any finely divided particles of matter so inhaled.

The carriage of disease-germs by ships' crews and cargoes, and the prevention of the introduction of contagious and infectious diseases by all the means and applications which should be grouped in the modern and scientific use of the term "maritime sanitation," have not escaped observation.

The pending national legislation to regulate inter-state commerce has a sanitary aspect that may in future prove of importance when there comes to the country the much desired national laws regulating "quarantine." Elsewhere I have cited the opinions of such jurists as Justices Marshall, Story and Grier, that "quarantine laws, health laws, etc., are regulations of commerce." How far the proposed inter-state commerce bill may make a classification of *goods* coming from infected places, or as to the *modes of conveyance*—i.e., railroad cars, canal boats, steamboats, etc., becoming carriers of disease-germs of themselves—or of infected goods, or in what manner this proposed *inter* (between and among) state laws may come in contact with *intra-* (within) state regulations of commerce, quarantine law, or health ordinances is unknown. As to *persons*, however, they are not the objects of commerce, and do not fall within the reasoning founded upon the construction of the power given by Congress, by the Constitution, to regulate commerce (City of New York *v.* Miln, 11 Peters, 102). What commercial regulations are to apply to a person suffering from a contagious or infectious disease in transit anywhere in the United States, among the States, cannot now be definitely formulated; within a State, that State's quarantine, health, and police laws now govern.

My own views upon this subject are of record, and the principles advanced have not changed even in the light of the recent International Conferences, but have been more decidedly fixed.

In the adaptation of the principles is the labor and toil.

